# Saffron Alters Microbial Amino Acid Metabolism and Neurotransmitter Production in a Defined Gut Consortium

**DOI:** 10.1002/fsn3.71694

**Published:** 2026-04-02

**Authors:** Adelaide E. Horvath, Makenna Grozis, Paul R. S. Baker, Santosh Kapil Kumar Gorti, Robert Proos, Ahmad Imran, Hassan Brim, Thomas D. Horvath, Melinda A. Engevik, Hassan Ashktorab

**Affiliations:** ^1^ Department of Regenerative Medicine & Cell Biology Medical University of South Carolina Charleston South Carolina USA; ^2^ Department of Biology & Biochemistry University of Houston Houston Texas USA; ^3^ Department of Mathematics University of Houston Houston Texas USA; ^4^ Department of Biology Elon University Elon North Carolina USA; ^5^ Metabolomics & Lipidomics Applications Team SCIEX Marlborough Massachusetts USA; ^6^ Department of Pathology and Cancer Center, College of Medicine Howard University College of Medicine Washington District of Columbia USA; ^7^ Department of Pathology & Immunology Baylor College of Medicine Houston Texas USA; ^8^ Department of Pathology Texas Children's Hospital Houston Texas USA; ^9^ Department of Pharmacy Practice & Translational Research, College of Pharmacy University of Houston Houston Texas USA; ^10^ Department of Pharmacology & Immunology Medical University of South Carolina Charleston South Carolina USA

**Keywords:** amino acids, bacterial communities, metabolism, neurotransmitters, saffron, short chain fatty acids

## Abstract

The gut microbiota relies on both dietary and host‐derived substrates to shape community composition, metabolic activity, and host physiology. While dietary fibers have been extensively studied as microbial substrates, less is known about how bioactive plant compounds influence microbial metabolism. The spice saffron, the stigma from the *Crocus sativus* flower, is commonly used for its medicinal traits, yet its effects on gut microbial communities remain poorly understood. Here, we used a defined human commensal consortium grown in anaerobic bioreactors to investigate how saffron alters bacterial metabolism. Saffron treatment significantly remodeled amino acid utilization and metabolite output, reducing tryptophan while increasing its downstream products tryptamine and indole acetic acid. In parallel, saffron elevated the levels of neuroactive compounds including GABA, glutamate, glycine, and dopamine, while decreasing L‐DOPA, tyrosine, and anthranilic acid. Short‐chain fatty acid (SCFA) profiles were also shifted, with increased formic and isobutyric acids, decreased propionic, butyric, and valeric acids, and no change in acetate, 2‐methyl‐butyric acid, and isovaleric acid. Together, these findings demonstrate that saffron profoundly reprograms microbial amino acid and neurotransmitter metabolism while reshaping SCFA production. This work provides new insight into how dietary bioactive compounds modulate microbial metabolic networks, with potential implications for gut and brain health.

## Graphical abstract

Graphical representation of the metabolic pathways modulated by saffron within defined microbial communities. In the tryptophan pathway, saffron decreased levels of tryptophan and anthranilic acid while increasing concentrations of indole‐3‐acetic acid and tryptamine. Within the tyrosine pathway, saffron reduced tyrosine and L‐DOPA levels and enhanced dopamine production. In the glutamate pathway, saffron increased concentrations of GABA, glutamate, and glutamine. Finally, in the short‐chain fatty acid and related metabolite category, saffron increased levels of isobutyric acid and formic acid, while decreasing propionic, butyric, and valeric acids.

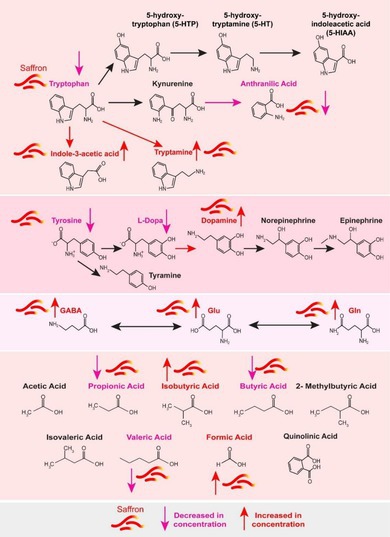

## 1 Introduction

The gastrointestinal tract harbors a dense and metabolically diverse microbial community that exerts profound effects on host physiology (Engevik and Engevik 2021; Engevik and Versalovic 2017; Contijoch et al. 2019). Beyond aiding in nutrient acquisition, gut microbes generate a wide array of metabolites that influence epithelial integrity, immune function, and systemic metabolic balance (Rowland et al. 2018). The availability of substrates, whether derived from diet or host secretions, shapes the composition of the microbiota and the metabolites produced (Feng et al. 2022). While dietary fibers have long been appreciated as microbial substrates, emerging evidence suggests that bioactive plant compounds also significantly influence microbial ecology and metabolism (Fu et al. 2022; Delzenne et al. 2025; Murga‐Garrido et al. 2021; Singh, Kaur, et al. 2025; Chen, Pan, et al. 2022).

Saffron, a spice derived from the stigma of *Crocus sativus* , has been traditionally used for its medicinal and neuroactive properties (El Midaoui et al. 2022; Kamalipour and Akhondzadeh 2011; Singletary 2020; Ashktorab et al. 2019). Preclinical and clinical studies have suggested roles for saffron and its bioactive components, such as crocin and safranal, in alleviating depression, anxiety, and inflammation (Chauhan et al. 2024; Bahrami et al. 2023; Dormal et al. 2025; Rashid et al. 2024). While these effects are often attributed to direct host interactions, growing evidence suggests that the gut microbiota may serve as a mediator of saffron's biological activity (Singh et al. 2023). However, knowledge regarding the extent to which saffron modulates microbial communities and the abundances and structural classes of microbial‐derived metabolites produced remains poorly defined.

A central feature of microbial metabolism is the transformation of dietary and host‐derived substrates into bioactive small molecules. Amino acids serve as key precursors for neuroactive metabolites with systemic consequences. For example, tryptophan can be converted into indoles, tryptamine, and serotonin precursors, many of which act on host receptors or signaling pathways (Horvath et al. 2023). Tyrosine metabolism yields L‐DOPA, dopamine, norepinephrine, and epinephrine, while glutamine can be converted into glutamate and subsequently into γ‐aminobutyric acid (GABA), a major inhibitory neurotransmitter (Horvath et al. 2023). In addition to amino acids, gut microbes produce short‐chain fatty acids (SCFAs) such as acetate, propionate, and butyrate as well as other organic acids like formate (Engevik and Versalovic 2017). Together, these metabolites have the potential to influence host metabolic and signaling pathways and thus represent a critical axis of host–microbe interaction.

Despite growing recognition of the gut microbiota's role in metabolite‐mediated signaling, little is known about how bioactive plant compounds such as saffron reprogram these metabolic networks. To address this gap, we employed a defined bacterial consortium representing major functional groups of the human gut microbiota. Using anaerobic bioreactors, we systematically profiled community composition and metabolite output following saffron treatment. We demonstrate that saffron profoundly alters amino acid metabolism, neurotransmitter‐related pathways, and SCFA production.

## 2 Methods

### 2.1 Saffron Preparation

We ground 20 mg of fresh saffron stigma (Amazon #B0BXFQHHQ1) in a marble mortar and pestle and then dissolved the powder in 1 mL of distilled water. The solution was incubated for 1 h on an orbital shaker in an aluminum foil wrapped container. We then filter sterilized the solution by running the solution through a 0.20 μm syringe filter. This solution was then immediately added to the bioreactors at a 2 mg/mL final concentration.

### 2.2 Designer Microbial Communities

To generate microbial communities that represented a reductionist version of the commensal human gut microbiota, we selected the following commercially available strains: *Akkermansia mucinphila* ATCC BAA83, *Clostridium symbiosum* DSZM 14940, *Bifidobacterium longum* ATCC 55813, *Lactococcus lactis* CB1, *Lactobacillus acidophilus* ATCC 4796, *Streptococcus thermophilus* ATCC 491, *Enterococcus faecalis* Symbioflor DSZM 16431, *Escherichia coli* Nissle DSZM 1917, *Prevotella copri* DSZM 18205, *Blautia coccoides* ATCC 29236, *Blautia producta* ATCC 27340D, *Bacteroides thetaiotaomicron* ATCC 29148, *Bacteroides fragilis* ATCC 23745, and *Bacteroides ovatus* ATCC 8483. These strains were selected because they have been identified in the human gut microbiota and they all have commensal properties (Ruan et al. 2020; Liu et al. 2022; Tingler and Engevik 2025; Lopetuso et al. 2013; Tingler et al. 2025; Harnvoravongchai et al. 2026; Lugli et al. 2025; Baccouri et al. 2019; Clare et al. 2024; Yeoh et al. 2022; Ihekweazu et al. 2021; Holmberg et al. 2024; Fultz et al. 2021; Gudi et al. 2026).

All bacteria were grown anaerobically overnight at 37°C in an Anaerobe Systems AS‐150 anaerobic chamber. Lactic acid bacteria *L. acidophilus, L* *. lactis* , *E. faecalis* , *S. thermophilus* , and *B. longum* were grown in Man, Rosa, Sharpe (MRS) medium, while *B. coccoides* , *B. producta* , *P. copri* , *C. symbiosum* , *E. coli* Nissle, *B. thetaiotaomicron* , *B. fragilis* , and *B. ovatus* were grown in brain heart infusion medium supplemented with 1% yeast extract and 0.1% cysteine (BHIS). *A. muciniphila* was grown in BHIS supplemented with 0.4% porcine gastric mucin. After confirming growth on a Motic AE2000 microscope, most cultures were centrifuged at 5,000*g* for 5 min to pellet bacteria. The exception was *A. muciniphila* , which was centrifuged at 9,000*g* for 5 min to pellet the bacteria. In each instance, bacterial pellets were washed 3× with sterile PBS to remove traces of the rich media. After the final wash, the bacterial pellet was resuspended in an equal volume of a chemically defined culture medium called ZMB1 (Horvath et al. 2023; Zhang et al. 2009; Engevik et al. 2023) and sub‐cultured to an optical density (OD_600nm_) of 0.05 in a 150 mL volume of ZMB1 in bioreactors (Engevik, Danhof, et al. 2021). Bioreactors (*n* = 6 in total) were randomly assigned to two groups: (1) vehicle (water) control and (2) saffron. To the bioreactors receiving saffron, we added 2 mg/mL final concentration of prepared saffron and to the control bioreactors we added the same volume of water. All cultures were grown in biological triplicate anaerobically at 37°C. After 72 h of incubation, cultures were centrifuged at 9000*g* for 5 min to pellet the bacteria for gDNA analysis and the cell‐free supernatants were sterile filtered using 0.2 μm syringe filters and processed for targeted metabolomics‐based bioanalysis. The bacterial pellets were processed for qPCR.

### 2.3 qPCR and Calculated CFUs

We isolated gDNA from the bacterial pellets using the Zymo Quick‐DNA Fecal/Soil Microbe Kits according to the manufacturer's instructions with bead beating. Quantitative real time PCR (qPCR) was performed using a Bio‐Rad CFX96 Real Time qPCR machine (Bio‐Rad). Forward and reverse primers for bacterial species were added to SYBR Green mastermix (Genesee Scientific #17‐501DP) and gDNA. Bacterial colony forming units (CFUs) were calculated from CT values based on standard curves of each bacteria (Ticer et al. 2024).

### 2.4 Quorum Sensing

To assess autoinducer‐2 (AI‐2)‐mediated quorum sensing, we employed the *Vibrio harveyi* bioluminescence assay using the MM32 reporter strain ( *V. harveyi* ATCC BAA‐1121, luxN::Tn5 luxS::Tn5), which responds exclusively to AI‐2 signals. *V. harveyi* was cultured overnight in Zobell marine broth at 30°C. Following overnight growth, cultures were adjusted to an OD_600nm_ of 1.0–1.1 and then were diluted 1:5000 in autoinducer medium and seeded into 96‐well plates. To each well, a 90 μL volume of the diluted *V. harveyi* culture in autoinducer medium was added and then a 10 μL volume of either cell‐free bioreactor supernatant or, as a control, uninoculated ZMB1 was added. For our controls, we used cell‐free supernatants from monocultures of *E. coli* K12 (positive control) and *E. coli* DHα (negative control). Plates were sealed, incubated at 30°C for 3 h, and subsequently monitored for luminescence in a Biotek Synergy H1 plate reader at 30°C with shaking at 175 rpm for an additional 3 h. Luminescence was recorded every 15 min, and induction was defined as the maximal difference between positive and negative controls, typically occurring between 3 and 5 h. Relative AI‐2 activity was calculated as the ratio of luminescence in experimental wells to that of negative controls.

### 2.5 LC–MS/MS Chemicals, Reagents, and Consumables

Optima LC/MS‐grade water, methanol, and acetonitrile were purchased from Fisher Scientific (Waltham, MA, USA). Mobile phase modifiers including MS‐grade ammonium formate and heptafluorobutyric acid were purchased from Millipore‐Sigma (Burlington, MA, USA). Authentic metabolite reference standards contained in the ZMBI growth media were purchased for the amino acids (alanine, arginine, asparagine, aspartate, cysteine, glutamine, glutamate, pyroglutamate, glycine, histidine, isoleucine, leucine, lysine, methionine, phenylalanine, proline, threonine, tryptophan, tyrosine, and valine), the vitamins (biotin, folic acid, nicotinamide, pantothenic acid, pyridoxine, riboflavin, and thiamine), the nucleic acids (adenine, guanine, uracil), sugars (glucose and inositol), and lipoic acid and glutathione were purchased from Millipore‐Sigma.

For the derivatization procedures used in the SCFA Method, the 1‐(3‐dimethylaminopropyl)‐3‐ethylcarbodiimide hydrochloride (EDAC), 2‐mercaptoethanol and succinic acid were each purchased from Fisher Scientific, and the aniline and [^13^C_6_]‐aniline (for carbon‐13 labeled internal standard (IS) standard synthesis) were each purchased from Millipore‐Sigma. For the preparation of the unlabeled analytical standards and the [^13^C_6_]‐labeled IS compounds, unlabeled Optima‐grade formic acid and acetic acid were purchased from Fisher Scientific, and unlabeled propionic acid, isobutyric acid, butyric acid, 2‐methylbutyric acid, isovaleric acid, valeric acid, and hexanoic acid reference standards were all purchased from Millipore‐Sigma. A 5‐μm Viva biphenyl (BiPh; 100 mm × 1 mm, 300 Å pore) analytical column and a 5‐μm Viva BiPh (10 mm × 2.1 mm) guard column were purchased from Restek (Bellefonte, PA, USA).

For the Glutamate Cycle Method, unlabeled GABA, L‐glutamate, and L‐glutamine analytical standards, and d6‐GABA, d5‐L‐glutamate, and d5‐L‐glutamine deuterated IS compounds, and a 2.7‐μm Supelco Ascentis Express HILIC (150 mm × 2.1 mm, 90 Å pore) analytical column were all purchased from Millipore‐Sigma.

For the Tryptophan Pathway Method, unlabeled N‐acetylserotonin and 5‐hydroxyindole‐3‐acetic acid (5‐HIAA) were purchased from Millipore‐Sigma, and unlabeled 5‐hydroxytryptophan, melatonin, serotonin hydrochloride, and L‐tryptophan analytical standards were all purchased from Fisher Scientific. Deuterated IS compounds including d5‐5‐HIAA, d3‐5‐hydroxytryptophan, d4‐melatonin, and d5‐L‐tryptophan were all purchased from CDN Isotopes (Pointe Claire, Quebec, Canada), and d4‐serotonin hydrochloride was purchased from Santa Cruz Biotechnology (Dallas, TX, USA). A 3‐μm Luna C18 (2) (150 mm × 1 mm, 100 Å pore) analytical column and a Security Guard C18 (4 mm × 2 mm) guard column were purchased from Phenomenex (Torrance, CA, USA).

For the Tyrosine Pathway Method unlabeled L‐tyramine was purchased from Fisher Scientific, and unlabeled anthranilic acid, dopamine hydrochloride, epinephrine, levodopa (L‐DOPA), D,L‐norepinephrine, and L‐tyrosine analytical standards were all purchased from Millipore‐Sigma. Deuterated IS compounds including d4‐dopamine, d6‐epinephrine and d3‐L‐DOPA were all purchased from Millipore‐Sigma, and d4‐L‐tyramine was purchased from Santa Cruz Biotechnology (Dallas, TX, USA). A 2.7‐μm Raptor C18 (100 mm × 1 mm, 90 Å pore) analytical column and a Restek Ultra C18 (10 mm × 2.1 mm, 100 Å pore) guard column were purchased from Restek.

### 2.6 SCIEX QTRAP 6500 (Classic)‐Based LC–MS/MS System (Targeted Metabolomics Methods)

The SCIEX QTRAP 6500 liquid chromatography–tandem mass spectrometry (LC–MS/MS) system used for the targeted bioanalysis consists of a Nexera X2 Ultrahigh‐Performance Liquid Chromatography (UHPLC) system (Shimadzu, Kyoto, Japan) connected to a QTRAP 6500 (Classic) hybrid triple‐quadrupole/linear ion trap mass spectrometer (SCIEX, Framingham, MA, USA). The system was operated using Analyst software (Version 1.6.2; SCIEX), while peak integration and quantitative analysis was performed using the MultiQuant software (Version 3.0.1; SCIEX). This system was used to perform the targeted bioanalysis for the SCFA Method, the Tyrosine Pathway and Tryptophan Pathway Methods, and the Glutamate Cycle Method for this project.

### 2.7 Targeted SCFA Method

The derivatization procedures and LC–MS/MS method conditions for the targeted SCFA Method have been described previously (Horvath et al. 2023, 2022; Engevik, Luck, et al. 2021; Luck et al. 2021). The final sample preparation procedure included the dilution of a 10 μL volume of derivatized bioreactor media sample in a 90 μL volume of an Internal Standard Solution‐A (ISS‐A) (DF = 37.4‐fold overall for each sample) that contained a concentration of 2500 nM each for the carbon‐13‐labeled IS compound derivatives including [^13^C_6_]‐N‐phenyl formamide, [^13^C_6_]‐N‐phenyl acetamide, [^13^C_6_]‐N‐phenyl propanamide, [^13^C_6_]‐N‐phenyl isobutanamide, [^13^C_6_]‐N‐phenyl butanamide, [^13^C_6_]‐N‐phenyl 2‐methylbutanamide, [^13^C_6_]‐N‐phenyl isopentanamide, [^13^C_6_]‐N‐phenyl pentanamide, and [^13^C_6_]‐N‐phenyl hexanamide in a solution of acetonitrile: water (2:8, vol/vol). A 4 μL volume of each sample was injected onto a QTRAP 6500‐based LC–MS/MS system for bioanalysis. The linear dynamic range for the method was 9.77–10,000 nM for each of the SCFA derivatives including N‐phenyl formamide, N‐phenyl acetamide, N‐phenyl propanamide, N‐phenyl isobutanamide, N‐phenyl butanamide, N‐phenyl 2‐methylbutanamide, N‐phenyl isopentanamide, N‐phenyl pentanamide, and N‐phenyl hexanamide.

### 2.8 Targeted Tyrosine Pathway Method

The critical solution preparations and LC–MS/MS conditions for the targeted Tyrosine Pathway Method have been described previously (Horvath et al. 2023, 2022; Engevik, Luck, et al. 2021; Luck et al. 2021). Briefly, a 10 μL volume of conditioned growth media was diluted in a 90 μL volume of an ISS‐A solution (DF = 10‐fold for each sample) that contained IS concentrations of 250 ng/mL for d_4_‐tyramine, 1000 ng/mL for d_3_‐L‐DOPA, 125 ng/mL for d_4_‐dopamine, and 200 ng/mL for d_6_‐epinephrine, and each sample was vortex‐mixed for ~30 s and transferred to an autosampler vial. A 10 μL volume of each sample was injected onto a QTRAP 6500‐based LC–MS/MS system for bioanalysis. The linear dynamic range for the method was 0.977–1000 ng/mL for the following unlabeled metabolites: tyramine, dopamine, L‐DOPA, tyrosine, norepinephrine, epinephrine, anthranilic acid, and quinolinic acid.

### 2.9 Targeted Tryptophan Pathway Method

The critical solution preparations and LC–MS/MS conditions for the targeted Tryptophan Pathway Method have been described previously (Horvath et al. 2023, 2022; Engevik, Luck, et al. 2021; Luck et al. 2021). Briefly, a 10 μL volume of conditioned growth media was diluted in a 90 μL volume of an ISS‐A solution (DF = 10‐fold for each sample) that contained IS concentrations of 500 ng/mL for d5‐tryptophan, d4‐serotonin and d4‐melatonin, and 1500 ng/mL for d5‐5‐HIAA, and each sample was vortex‐mixed for ~30 s and transferred to an autosampler vial. A 10 μL volume of each sample was injected onto a QTRAP 6500‐based LC–MS/MS system for bioanalysis. The linear dynamic range for the method was 0.977–1000 ng/mL for the following unlabeled metabolites: tryptophan, serotonin, melatonin, 5‐HIAA, 5‐hydroxytryptophan, N‐acetylserotonin, tryptamine, and indoleacetic acid.

### 2.10 Targeted Glutamate Cycle Method

The critical solution preparations and LC–MS/MS conditions for the targeted Glutamate Cycle Method have been described previously (Horvath et al. 2023, 2022; Engevik, Luck, et al. 2021; Luck et al. 2021). Briefly, a 10 μL volume of conditioned growth media was diluted in a 90 μL volume of an ISS‐A solution (DF = 10‐fold for each sample) that contained IS concentrations of 500 ng/mL for d6‐GABA, d5‐glutamate and d5‐glutamine, and each sample was vortex‐mixed for ~30 s and transferred to an autosampler vial. A 10 μL volume of each sample was injected into a QTRAP 6500‐based LC–MS/MS system for bioanalysis. The linear dynamic range for the method was 0.977–1000 ng/mL for the following unlabeled metabolites: GABA, glutamate and glutamine.

### 2.11 Critical Solution Preparations for the Individual ZMBI Components for the Quasi‐Targeted Metabolomics (QT‐Meta) Method

The LC–MS/MS‐based QT‐Meta Method described here is based on a commercially‐developed SCIEX Method (RUO‐MKT‐02‐12750‐A) that was bundled with the QTRAP 7500 purchase. To ensure complete coverage of the metabolites used in ZMBI growth medium preparations, the molecule‐specific multiple‐reaction monitoring (MRM) parameters were optimized for each ZMBI component in positive and negative ion modes on the QTRAP 7500 MS system; all other metabolites included in the SCIEX QT‐Meta Method were excluded from the acquisition method because we were primarily interested in microbial consumption of the ZMBI components for this study.

Individual Stock Solutions for each ZMBI media component listed in the Chemicals, Reagents, and Consumables Section above were each prepared at solution concentrations of 10 mg/mL in solvent systems described previously (Engevik et al. 2023), and all Stock Solutions were vortex‐mixed briefly. Individual Intermediate Solutions were prepared for each ZMBI component at solution concentrations of 100 μg/mL by diluting a 10 μL volume of the respective Stock Solution in a 990 μL volume of methanol: water (1:1, *v:v*) solution, and all Intermediate Solutions were vortex‐mixed briefly. Individual Infusion Solutions were prepared for each ZMBI component at solution concentrations of 500 ng/mL by diluting a 5 μL volume of the respective Intermediate Solution in a 995 μL volume of methanol: water (1:1, *v:v*) solution, and all Infusion Solutions were vortex‐mixed briefly. *Technical Note: if the precursor ion signal was found to be too intense in the prepared infusion solutions (≥ 7e+7 counts/second in intensity), then the tuning solution was further diluted 5–10‐fold directly in the infusion syringe by the addition of an appropriate volume of a neat methanol: water (1:1, v:v) solution*.

### 2.12 SCIEX QTRAP 7500 (Classic)‐Based LC–MS/MS System (Quasi‐Targeted Metabolomics Method)

The UHPLC–MS/MS system was comprised of a Shimadzu Nexera 40 Series UHPLC system outfitted with a SIL‐30ACMP autosampler (Kyoto, Japan) coupled to a SCIEX QTRAP 7500 (Classic) hybrid triple quadrupole/linear ion trap mass spectrometer using the SCIEX OS (Ver. 3.3.1.43) software for operational control and to perform relative quantitation.

### 2.13 Metabolite Optimizations on the LC–MS/MS System for the QT‐Metabolomics Method

Molecule‐specific tuning of the MS system for each of individual ZMBI components was performed using the MS Method module in SCIEX OS. A volume of ~1 mL of the respective metabolite Infusion Solution was drawn into a 1 mL Gastight (#1001; P/N: 81320) syringe by Hamilton (Reno, Nevada). Each metabolite Infusion Solution was infused into the ionization source of the MS system at a flowrate of 10 μL/min and the mass‐to‐charge (*m/z*) and precursor ions (i.e., [M + H]+ or [M − H]−) were determined for each metabolite using a Q1 based precursor ion scan with Start mass (Da) and Stop mass (Da) of ± *m/z* 20 bracketing the theoretical precursor ion (*m/z*) for the metabolite being examined. Entrance potential (EP) voltages were not optimized for each metabolite but were set to +15 V or −15 V for positive and negative modes, respectively, for all metabolites. A Q3 Product Ion spectrum was acquired for each metabolite (at the appropriate precursor ion *m/z for each metabolite*) by ramping the collision energy (CE) using the Ramp Compound Parameter Function with Start and Stop CEs of +5 to +80 eV for positive mode, or −5 eV and −80 eV for negative mode, respectively, using a 2 eV step size in each instance. Following the Q3 product ion scan, an MRM scan was performed to optimize the CE for the top 4–5 most intense product ions for each metabolite, and the top 2–3 were selected for inclusion in the acquisition method. Collision‐cell exit potential (CXP) voltages weren't optimized for each metabolite but rather were set to +15 V or −15 V for positive and negative modes, respectively, for all metabolites.

### 2.14 LC–MS/MS Method for the QT‐Meta Method

Chromatographic separations were performed using a Kinetex 2.6‐μm F5 (150 mm x 2.1 mm, 100 Å; cat #: 00F‐4723‐AN) analytical column with an attached SecurityGuard F5 (2.1 mm; cat #: AJO‐9322) Ultra Cartridge that was both purchased from Phenomenex (Torrance, CA, USA). Chromatographic separations were performed using mobile phase A (MPA) and a mobile phase B (MPB) solutions consisting of mixtures of 0.1% formic acid in water, and 0.1% formic acid in acetonitrile, respectively. The needlewash solution consisted of a mixture of water: methanol: isopropanol: acetonitrile (1: 1: 1: 1, *v: v: v: v*). These solutions were stored sealed at ambient temperature and expired 1 month after preparation. Operational parameters for the UHPLC system included a mobile phase flowrate of 0.200 mL/min, an autosampler sample bay chilling temperature of +6°C, a column oven heating temperature of +30°C, and a gradient elution program specified as follows: 0.0–2.1 min, 0% MPB; 2.1–14.0 min, 0%–95% MPB; 14.0–16.0 min, 95% MPB; 16.0–16.1 min, 95%–0% MPB; and 16.1–20.0 min, 0% MPB, with a gradient cycle time of approximately 20.4 min per sample.

A High‐flow (> 200 μL/min chromatographic flowrate) TurboIonSpray electrospray ionization (ESI) probe and an E‐Lens orthogonal probe were each installed in the OptiFlow Pro Ionization Source that was attached to the inlet of the QTRAP 7500 MS system. After the MRM optimizations for each of the metabolites was completed, individual injections of a 5 μL volume of aqueous metabolite standards (500 ng/mL) were injected onto the UHPLC–MS/MS system, using the Batch, Queue, and Explore modules in SCIEX OS, in order to empirically determine the RTs for each metabolite using the chromatographic system described. The metabolite RTs were used to create a sMRM‐based scanning method that used positive and negative mode polarity switching with Settling and Pause times of 5 ms each specified, respectively. Ionization source parameters were specified as follows: Ion source gas 1, 30 pounds per square inch (PSI); Ion source gas 2, 50 PSI; Curtain gas, 40 PSI; Collisionally‐activated dissociation (CAD) gas, 9 (arbitrary); Source temperature, +350°C; Positive mode ionspray (IS) voltage, +3500 V; Negative mode IS voltage, −3500 V; Apply sMRM triggering, off; and, Q0 dissociation, off.

Triplicate injections of a blank ZMBI culture media were made in order to create a peak integration method using the Analytics module of SCIEX OS—this method was created specifically to monitor for microbial metabolism of ZMBI components during microbial growth from inoculation to log‐phase. Peak integration parameters were optimized for the integration of each metabolite contained in the blank ZMBI culture media in the presence of the other media compounds—this quantitation method file was saved so that it may be used to integrate metabolite peaks present in bacterial‐conditioned culture media.

### 2.15 Bacterial‐Conditioned Media Sample Preparations

Prior to analysis, all blank and cell‐free bacterial‐conditioned ZMBI media samples were thawed on a benchtop at ambient temperature, and were then vortex‐mixed for 30 s to ensure thorough mixing prior to dilution. Then in a glass autosampler vial, a 2 μL volume of each cell‐free media sample was diluted in a 998 μL volume of a dilution solution consisting of water: acetonitrile: formic acid (95: 5: 0.1, *v: v: v*), for a 500‐fold dilution overall. Then a 2 μL volume of diluted sample was injected onto the LC–MS/MS system for analysis. The quantitation method filename was specified in the batch file so that the integration of each metabolite peak could be automatically integrated after the completion of data acquisition for each blank and media sample.

### 2.16 Graphs and Statistical Analysis

All graphs and statistical analyses were performed using GraphPad Prism (version 10.03) software (GraphPad Inc., La Jolla, CA). Comparisons were made with either a One‐way or Two‐way Analysis of Variance (ANOVA) with the Bonferroni post hoc test or student *t*‐test for data with only two groups. Non‐parametric data were log‐transformed to pass normality tests before analysis by ANOVA. Differences between the groups were considered significant at *p* < 0.05 (*) and the data are presented as mean ± standard deviation.

## 3 Results

**FIGURE 1:**
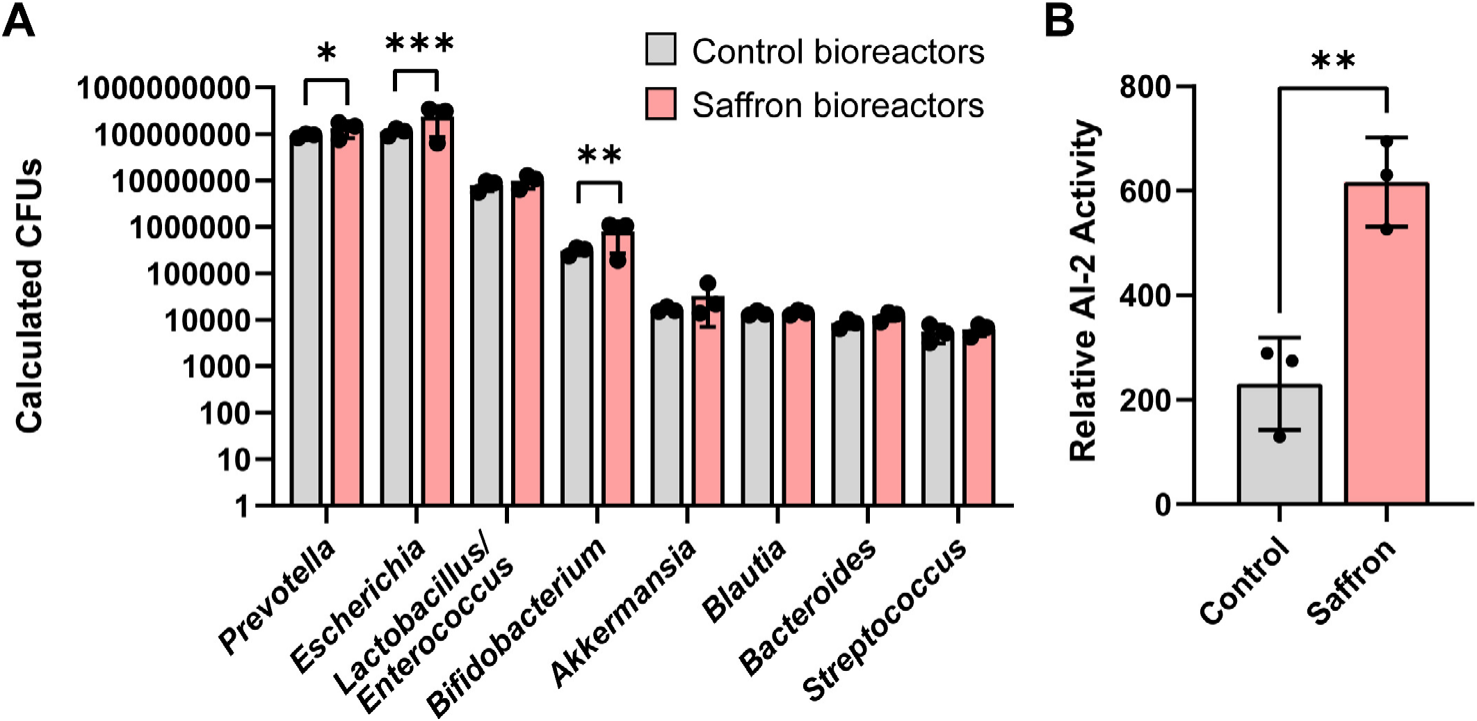
(A) Quantification of bacterial growth in defined microbial bioreactors. Colony‐forming units (CFUs) were estimated by qPCR targeting the genera specific genes and converted to cell equivalents using standard curves generated from known bacterial titers. CFUs are shown for control (gray bars) and saffron‐treated (pink bars) bioreactors. (B) Relative autoinducer‐2 (AI‐2) quorum sensing activity was quantified using the *Vibrio harveyi* MM32 bioluminescence assay from cell‐free supernatants from bioreactors (control and saffron‐treated). Data represent mean ± standard deviation of biological replicates (*n* = 3/group). Statistical significance was determined using ANOVA (A) and *t*‐test (B). Each dot represents an individual sample, and the error bars represent the standard error of the mean. *p* < 0.05 (*), *p* ≤ 0.01 (**), *p* ≤ 0.001 (***), *p* ≤ 0.0001 (****).

To investigate how saffron influences gut microbial communities, we established a reductionist model of the human commensal microbiota using a defined consortium of 14 representative bacterial species. This community included *Akkermansia muciniphila* , *Clostridium symbiosum* , *Bifidobacterium longum* , *Lactococcus lactis* , *Lactobacillus acidophilus* , *Streptococcus thermophilus* , *Enterococcus faecalis* Symbioflor, *Escherichia coli* Nissle, *Prevotella copri* , *Blautia coccoides* , *Blautia producta* , *Bacteroides thetaiotaomicron* , *Bacteroides fragilis* , and *Bacteroides ovatus* . This defined microbial community recapitulates major functional groups of the human gut microbiota (Ruan et al. 2020), enabling controlled analysis of community dynamics, metabolite production, and signaling in response to dietary interventions. We grew these bacteria together in anaerobic bioreactors, and after 3 days of growth we assessed the bacterial community (Figure 1A). The addition of 2 mg/mL saffron increased microbial growth (Control: OD_600nm_ = 13.3 ± 0.6, calculated CFUs = 1.8 × 10^8^ ± 2.7 × 10^7^; Saffron: OD_600nm_ = 14.4 ± 0.2, calculated CFUs = 3.9 × 10^8^ ± 3.1 × 10^7^; *p* = 0.01) with significant expansions in *Prevotella*, *Escherichia*, and *Bifidobacterium* species compared to control bioreactors (Figure 1A). These data suggest that saffron promotes overall bacterial growth while selectively enriching specific taxa.

Since saffron increased the abundance of several community members, we next asked whether it also influenced microbial communication. Many gut bacteria coordinate group behaviors through quorum sensing, and the universal signaling molecule autoinducer‐2 (AI‐2) has been proposed to mediate interspecies communication. Using the *Vibrio harveyi* MM32 reporter assay, which selectively identifies AI‐2 signaling, we found that saffron supplementation significantly enhanced the production of AI‐2 (Figure 1B). These findings suggest that saffron alters the composition of the microbial community and enhances microbial communication through quorum sensing pathways.

**FIGURE 2:**
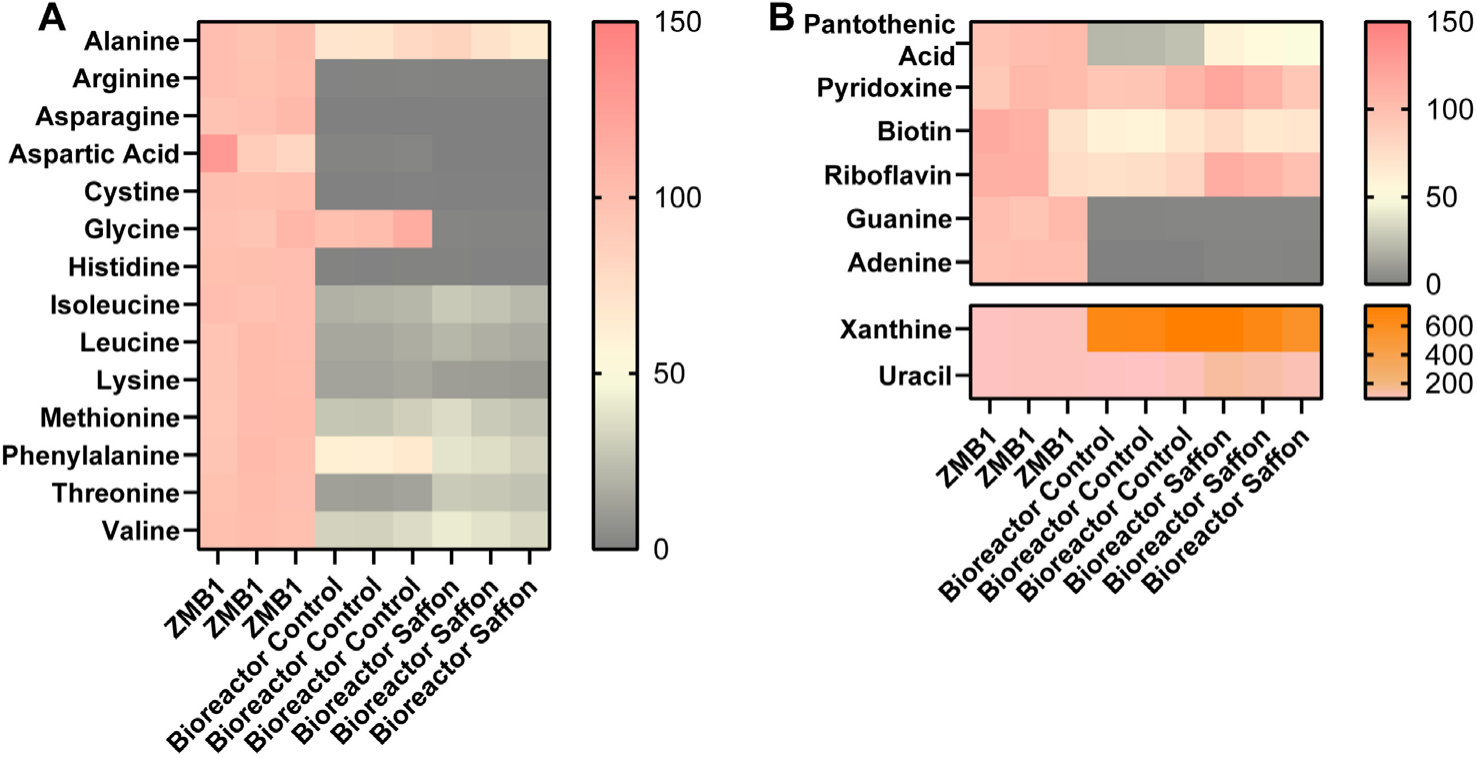
(A) Heat map of the percentage of amino acids in uninoculated ZMB1, control bioreactors, and saffron supplemented bioreactors as assessed by LC–MS/MS. (B) Heat map of the percentage of other compounds, including vitamins and nucleic acids, in uninoculated ZMB1, control bioreactors, and saffron supplemented bioreactors as assessed by LC–MS/MS (*n* = 3/group). See Table 1 for statistical analysis.

Since our bacterial medium, ZMB1, was chemically defined, we examined the major components of the medium consumed by the bioreactor communities. Both control and saffron‐treated communities depleted multiple amino acids, including arginine, asparagine, aspartate, cystine, histidine, isoleucine, leucine, lysine, methionine, phenylalanine, threonine, and valine (Figure 2A, Table 1). Among these amino acids, saffron‐treated communities showed lower levels of glycine and phenylalanine compared to controls, but did not reduce threonine to the same extent, suggesting an altered amino acid consumption profile. We also examined the utilization of vitamins and nucleotides included in the ZMBI medium. Both control and saffron‐treated bioreactors reduced pantothenic acid, biotin, guanine, and adenine, while elevating xanthine and uracil (Figure 2B). Interestingly, control communities significantly reduced riboflavin compared to blank medium, whereas saffron‐treated communities did not, suggesting that saffron preserves riboflavin availability (Figure 2B).

**TABLE 1:** Consumption or production of ZMB1 medium components by defined microbial communities. The table depicts the average percentage ± standard deviation of individual ZMB1 medium components, including amino acids, nucleobases, and vitamins, as measured by LC–MS/MS. The uninoculated ZMB1 medium was normalized to 100% for each compound and used as the reference condition. Values shown for control and saffron‐treated bioreactors represent the relative percentage of each medium component remaining after growth of the defined microbial communities, reflecting microbial utilization or production. Left columns indicate the *p*‐values for comparisons between groups determined by two‐way ANOVA (*n* = 3 bioreactors per group).

Compound	ZMB1 medium average ± SD	Control bioreactors average ± SD	Saffron treated bioreactors average ± SD	ZMBI vs control bioreactors *P*	ZMB1 vs saffron treated bioreactor *P*	Control bioreactors vs saffron bioreactors *P*
Alanine	100.0 ± 2.5	73.0 ± 5.5	73.8 ± 9.0	< 0.0001	< 0.0001	0.9821
Arginine	100.0 ± 1.8	1.1 ± 0.2	0.8 ± 0.0	< 0.0001	< 0.0001	0.9967
Asparagine	100.0 ± 4.0	0.0 ± 0.1	0.0 ± 0.0	< 0.0001	< 0.0001	> 0.9999
Aspartic acid	99.9 ± 25.9	1.6 ± 0.5	0.0 ± 0.0	< 0.0001	< 0.0001	0.922
Cystine	100.0 ± 1.1	0.4 ± 0.2	0.1 ± 0.0	< 0.0001	< 0.0001	0.9967
Glycine	100.0 ± 5.5	105.2 ± 8.5	1.4 ± 0.2	0.4284	< 0.0001	< 0.0001
Histidine	100 ± 0.6	0.8 ± 0.1	0.7 ± 0.1	< 0.0001	< 0.0001	0.9999
Isoleucine	100.0 ± 1.4	19.9 ± 1.1	25.6 ± 3.2	< 0.0001	< 0.0001	0.3569
Leucine	100.0 ± 3.6	15.8 ± 1.4	18.7 ± 2.3	< 0.0001	< 0.0001	0.7661
Lysine	100.0 ± 3.8	14.1 ± 0.8	11.1 ± 0.7	< 0.0001	< 0.0001	0.7491
Methionine	100.0 ±	28.4 ± 2.3	30.4 ± 5.1	< 0.0001	< 0.0001	0.8808
Phenylalanine	100.0 ± 4.7	63.0 ± 2.6	36.0 ± 3.6	< 0.0001	< 0.0001	< 0.0001
Threonine	100.0 ± 2.2	12.8 ± 0.7	27.5 ± 1.7	< 0.0001	< 0.0001	0.0019
Valine	100.0 ± 1.5	33.4 ± 2.3	38.1 ± 4.0	< 0.0001	< 0.0001	0.4904
Pantothenic Acid	100.0 ± 3.6	23.0 ± 1.9	53.7 ± 5.5	< 0.0001	< 0.0001	0.0006
Pyridoxine	100.0 ± 7.1	98.9 ± 6.9	107.0 ± 13.2	0.9873	0.6132	0.5192
Biotin	100.0 ± 23.8	62.8 ± 5.7	71.9 ± 5.7	< 0.0001	0.0016	0.4385
Riboflavin	100.0 ± 20.6	76.9 ± 4.5	107.2 ± 7.8	0.01	0.5964	0.0007
Guanine	100.0 ± 4.4	1.9 ± 0.1	2.2 ± 0.1	< 0.0001	< 0.0001	0.9994
Adenine	100.0 ± 1.5	0.3 ± 0.0	1.6 ± 0.2	< 0.0001	< 0.0001	0.9833
Xanthine	100.0 ± 4.5	683.9 ± 49.6	649.1 ± 87.5	< 0.0001	< 0.0001	0.5803
Uracil	100.0 ± 3.6	96.8 ± 6.8	130.6 ± 17.4	0.9952	0.6538	0.5977

**FIGURE 3:**
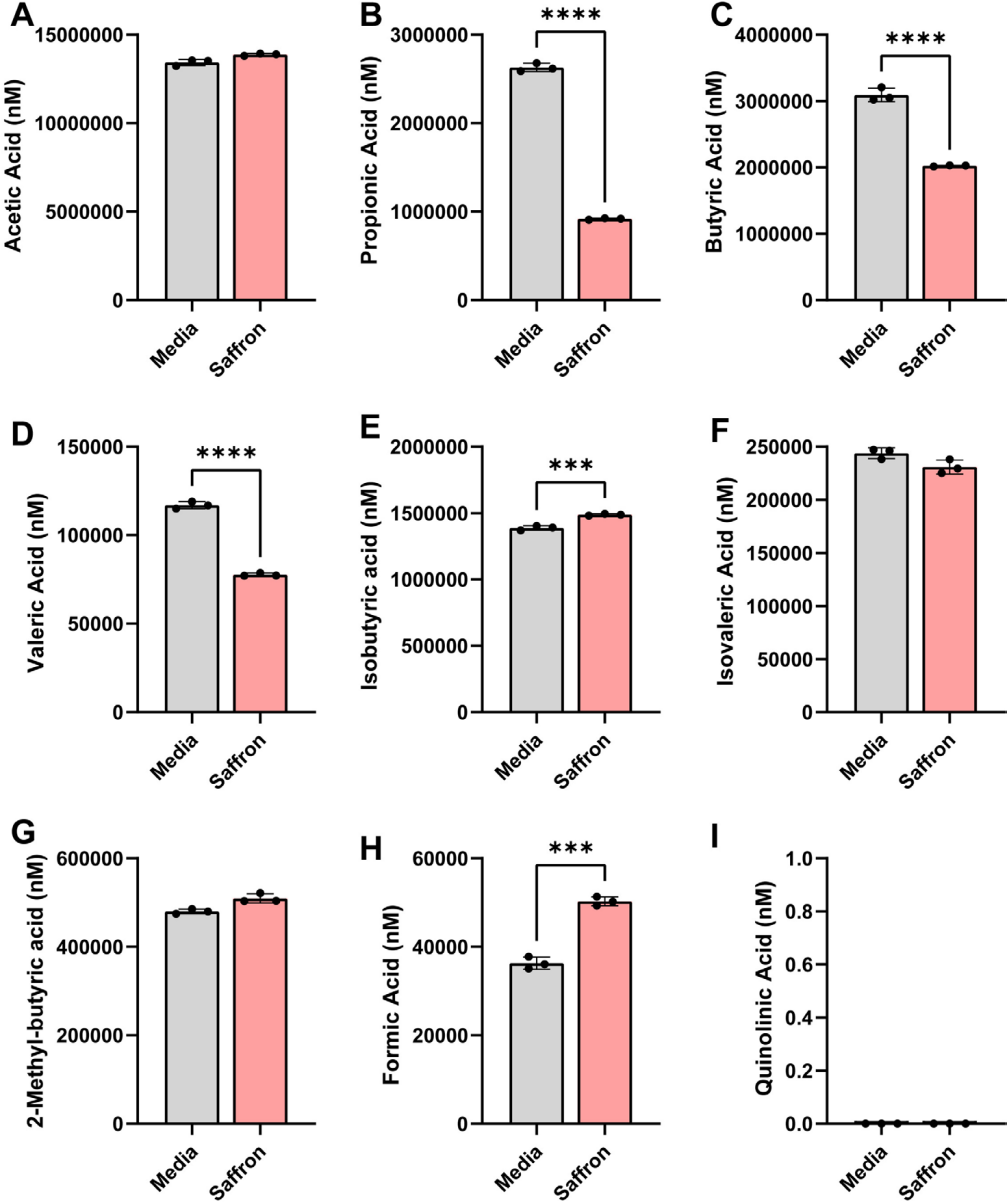
Individual bar graphs depicting the concentrations (nM) of short chain fatty acids (SCFAs), formic acid, and quinolinic acid in cell‐free supernatant from control (gray) and saffron (pink) supplemented bioreactors as measured by targeted LC–MS/MS. Bar graphs represent the following compounds: (A) Acetic acid (acetate), (B) propionic acid (propionate), (C) butyric acid (butyrate), (D) valeric acid (valerate), (E) isobutyric acid (isobutyrate), (F) isovaleric acid (isovalerate), (G) 2‐methyl‐butyric acid (2‐methyl butyrate), (H) formic acid (formate), and (I) quinolinic acid. Quinolinic acid was undetectable in our microbial communities. Analyzed by Students *T*‐test. *p* < 0.05 (*), *p* ≤ 0.01 (**), *p* ≤ 0.001 (***), *p* ≤ 0.0001 (****). Each dot represents data from an individual bioreactor (*n* = 3/group) and each error bar represents the standard deviation (*n* = 3/group).

To determine how saffron impacted microbial fermentation, we examined the levels of microbial‐produced SCFAs by targeted LC–MS/MS (Figure 3A–I). Saffron significantly decreased the concentrations of propionic acid, butyric acid, and valeric acid (Figure 3B–D), while increasing the levels of isobutyric acid (Figure 3E). We did not observe changes in acetic acid, isovaleric acid, or 2‐methylbutyric acid between bioreactor communities (Figure 3A,F,G). We also measured formic acid and quinolinic acid because both metabolites are closely linked to microbial energy and redox metabolism (Sawers 2025). Saffron supplementation increased the levels of formic acid (Figure 3H), while quinolinic acid was undetectable in our microbial communities (Figure 3I). These data indicate that saffron supplementation promotes the generation of isobutyric and formic acid.

**FIGURE 4:**
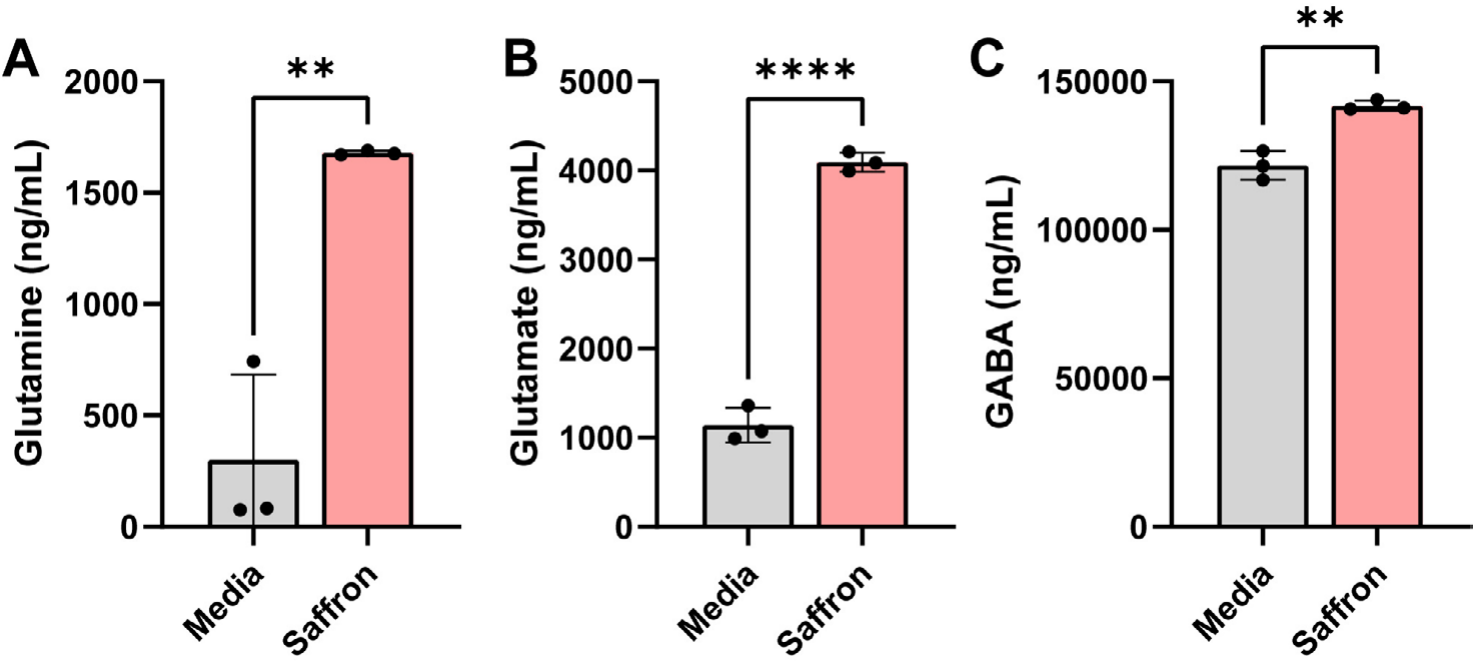
Individual bar graphs depicting the concentrations (ng/mL) of compounds in the Glu/Gln/GABA pathway in cell‐free supernatant from control (gray) and saffron (pink) supplemented bioreactors as measured by targeted LC–MS/MS. Bar graphs represent the following compounds: (A) Glutamine, (B) Glutamate, and (C) GABA. Analyzed by students *T*‐test. *p* < 0.05 (*), *p* ≤ 0.01 (**), *p* ≤ 0.001 (***), *p* ≤ 0.0001 (****). Each dot represents data from an individual bioreactor (*n* = 3) and each error bar represents the standard deviation. Analyzed by student *T*‐test. *p* < 0.05 (*), *p* ≤ 0.01 (**), *p* ≤ 0.001 (***), *p* ≤ 0.0001 (****) (*n* = 3/group).

We next asked whether saffron altered the production of neurotransmitters and neuroactive metabolites/precursors. We first examined the glutamine/glutamate/GABA cycle (Figure 4A–C). In this pathway, glutamine can be converted into glutamate, glutamate can be converted into glutamine, or glutamate can subsequently be converted into GABA (Horvath et al. 2023). Additionally, some microbes can de novo synthesize glutamine and glutamate (Yan 2007; Helling 1998; Kim Jong et al. 2012), thereby elevating the levels of these amino acids. In saffron‐treated bioreactors, we observed significant increases in glutamine, glutamate, and GABA compared to controls. These data suggest that saffron enhances the microbial capacity to generate neuroactive metabolites within this pathway.

**FIGURE 5:**
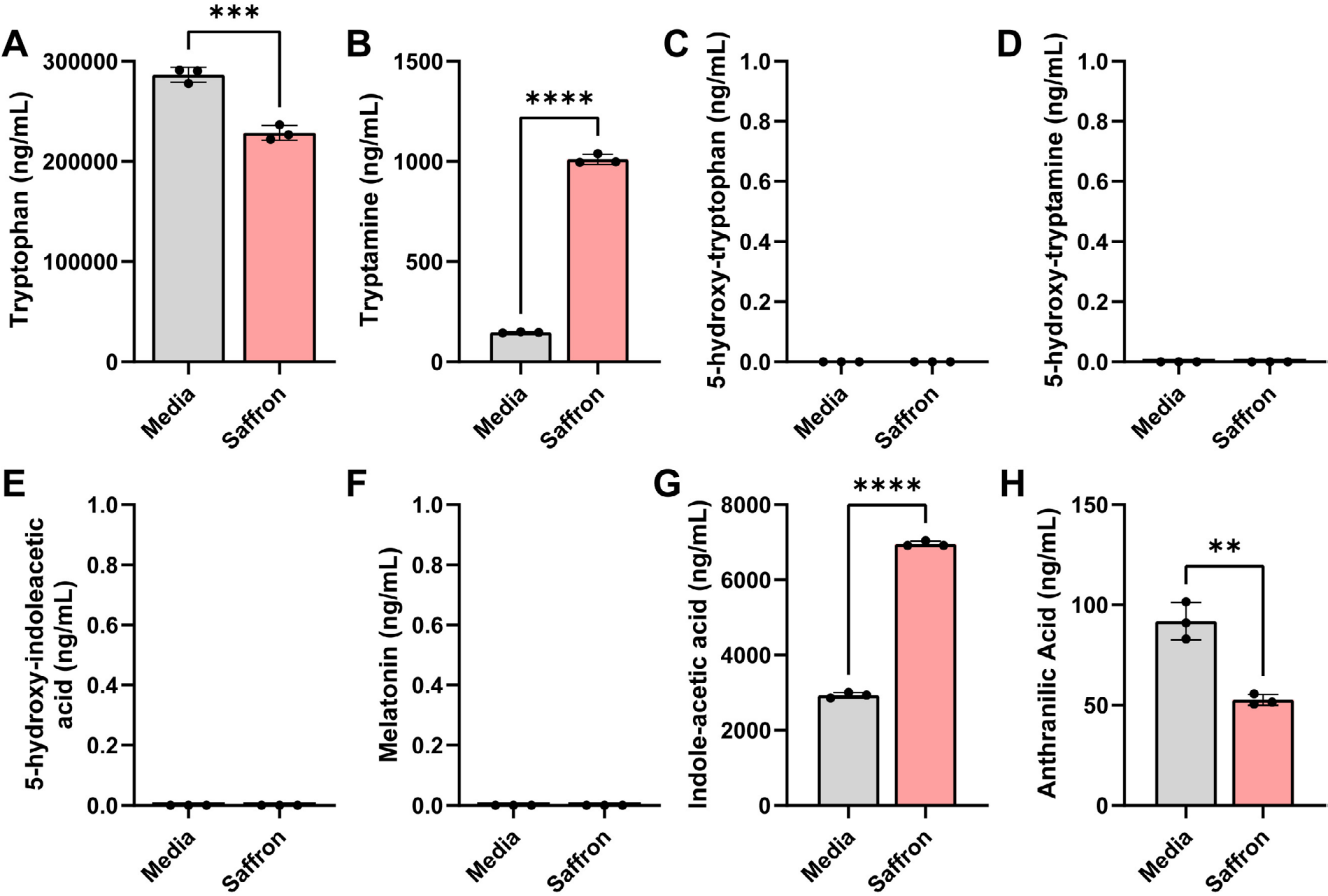
Individual bar graphs depicting the concentrations (ng/mL) of compounds in the tryptophan pathway in cell‐free supernatant from control (gray) and saffron (pink) supplemented bioreactors as measured by targeted LC–MS/MS. Bar graphs represent the following compounds: (A) Tryptophan, (B) Tryptamine, (C) 5‐hydroxytryptophan (5‐HTP), (D) 5‐hydroxytryptamine (5‐HT, serotonin), (E) 5‐hydroxy‐indole acetic acid (5‐HIAA), (F) melatonin, (G) indole acetic acid, and (H) anthranilic acid. 5‐HTP, 5‐HT, 5‐HIAA, and melatonin were undetectable in our microbial communities. Analyzed by students *T*‐test. *p* < 0.05 (*), *p* ≤ 0.01 (**), *p* ≤ 0.001 (***), *p* ≤ 0.0001 (****). Each dot represents data from an individual bioreactor (*n* = 3) and each error bar represents the standard deviation. One ANOVA, *p* < 0.05 (*), *p* ≤ 0.01 (**), *p* ≤ 0.001 (***), *p* ≤ 0.0001 (****) (*n* = 3/group).

We then examined the tryptophan pathway (Figure 5A–H). In this pathway, tryptophan can be converted into tryptamine, which can activate serotonin receptors in the gut (Bhattarai et al. 2018), or into 5‐hydroxytryptophan (5‐HTP) and subsequently serotonin (5‐hydroxy‐tryptamine, 5‐HT; Horvath et al. 2023). Serotonin can then be degraded into 5‐hydroxyindoleacetic acid (5‐HIAA) or converted into melatonin (Horvath et al. 2023). Alternatively, tryptophan can be degraded by some bacteria into indoles, such as indole‐3‐acetic acid, or into kynurenine, which can be converted into anthranilic acid (Ihekweazu et al. 2021). We found that saffron treatment significantly reduced tryptophan levels (Figure 5A) and elevated tryptamine (Figure 5B). We did not detect any 5‐hydroxy‐tryptophan (5‐HTP), 5‐hydroxy‐tryptamine (5‐HT), 5‐hydroxyindoleacetic acid (5‐HIAA), or melatonin in any of our bioreactors (Figure 5C–F), suggesting that none of our bacteria were able to generate these compounds. We also found that saffron treatment elevated the production of indole‐3‐acetic acid and reduced anthranilic acid levels (Figure 5G,H). These findings suggest that saffron drives tryptophan metabolism toward tryptamine and indole derivatives while suppressing anthranilate production.

**FIGURE 6:**
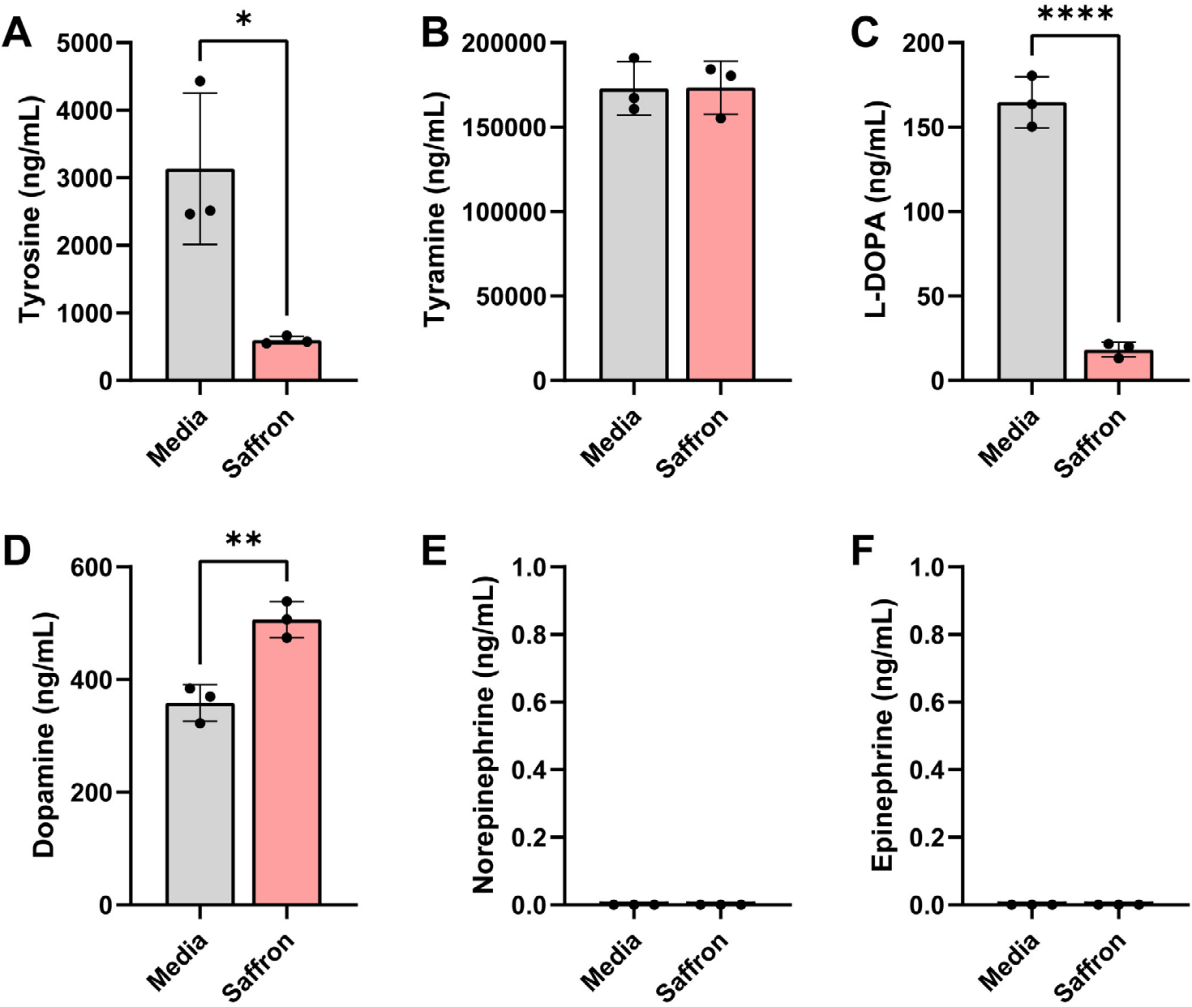
Individual bar graphs depicting the concentrations (ng/mL) of compounds in the tyrosine pathway in cell‐free supernatant from control (gray) and saffron (pink) supplemented bioreactors as measured by targeted LC–MS/MS. Bar graphs represent the following compounds: (A) Tyrosine, (B) Tyramine, (C) L‐DOPA, (D) Dopamine, (E) Norepinephrine, and (F) Epinephrine. Norepinephrine and epinephrine were undetectable in our microbial communities. Analyzed by student *T*‐test. *p* < 0.05 (*), *p* ≤ 0.01 (**), *p* ≤ 0.001 (***), *p* ≤ 0.0001 (****). Each dot represents data from an individual bioreactor (*n* = 3) and each error bar represents the standard deviation. Analyzed by student *T*‐test. *p* < 0.05 (*), *p* ≤ 0.01 (**), *p* ≤ 0.001 (***), *p* ≤ 0.0001 (****) (*n* = 3/group).

In the tyrosine pathway, tyrosine can be converted to L‐DOPA and subsequently to dopamine. Dopamine can in turn be converted into norepinephrine and then epinephrine (Horvath et al. 2023). We found that saffron significantly decreased tyrosine and L‐DOPA levels (Figure 6A,C), but did not affect the levels of tyramine (Figure 6B). Interestingly, we found that saffron markedly elevated dopamine concentrations in our bioreactor communities (Figure 6D). We did not find detectable levels of norepinephrine or epinephrine in the bioreactors (Figure 6E,F). These results suggest that saffron promotes conversion of tyrosine metabolites into dopamine. Collectively, this study indicates that saffron can significantly influence both the composition and function of the human gut microbiota in a reductionist model.

## 4 Discussion

In this study, we developed a defined, reductionist model of the human gut microbiota to investigate how saffron shapes microbial community structure and function. Using a 14‐member consortium representing diverse commensal taxa, we show that saffron supplementation increased the overall bacterial abundance, enhanced AI‐2 signaling, and increased the concentration of isobutyric acid. In terms of amino acids, we found that saffron‐treated bacterial communities depleted more glycine and phenylalanine compared to vehicle control‐treated bacterial communities. Moreover, saffron shifted aromatic amino acid metabolism, elevating dopamine, tryptamine, and indole acetic acid. These findings suggest that saffron promotes microbial communication and alters the production of both metabolic and neuroactive compounds in defined microbial communities.

One of the interesting findings of our study was that saffron significantly impacted the gut microbiota and increased *Prevotella* presence within the model. This aligns with previous studies that demonstrate that saffron can alter the gut microbiota in mice (Singh et al. 2023; Peng et al. 2023; Chen, Wang, et al. 2022; Banskota et al. 2021; Pontifex et al. 2022), rats (Güllü et al. 2019), and humans (Lang et al. 2025; García et al. 2024). For example, Pontifex et al. identified that saffron supplementation increased *Alloprevotella* in mice and Lang et al. found that saffron supplementation in adults elevated commensal *Prevotella* (Lang et al. 2025). *Prevotella* species in the human gut are primarily comprised of *P. copri* (Yeoh et al. 2022; Abdelsalam et al. 2023); the organism that we used in this study. *Prevotella* species have been correlated with the consumption of plant fiber and polyphenol rich diets (Precup and Vodnar 2019; Pareek et al. 2019; Wang et al. 2022; Kovatcheva‐Datchary et al. 2015). Kovatcheva‐Datchary et al. found that people who responded to a high fiber diet with improved glucose metabolism had high levels of *Prevotella* and when fecal specimens collected from these individuals were transplanted into germ‐free mice, the microbiota from responder human donors exhibited improved glucose metabolism and increased abundance of *Prevotella* (Kovatcheva‐Datchary et al. 2015). These findings indicate that *Prevotella* might be a saffron sensitive beneficial microbe. Although saffron is not a significant source of dietary fiber, it is rich in polyphenols (Slimani et al. 2025). Saffron‐associated polyphenols, including crocin, crocetin, and safranal, may act as substrates or signaling molecules that favor the growth of *Prevotella* and other commensals. Our findings suggest that saffron provides substrates or ecological advantages that favor *Prevotella* expansion, consistent with emerging evidence that phytochemicals can act as growth modulators for particular commensal bacteria (Jit et al. 2021). Future studies will be needed to directly test how *Prevotella* species respond to saffron and its bioactive components.

We also found that saffron increased the abundance of *Bifidobacterium*. Many *Bifidobacterium* species, including *B. longum, B. bifidum, B. dentium, B* *. infantis* , and *B. breve* , have been documented to benefit the host (Engevik, Luck, et al. 2021; Engevik et al. 2019; Luck et al. 2020; Di Gioia et al. 2014; Khailova et al. 2009; Zhang et al. 2019; Fukuda et al. 2011). Most *Bifidobacterium* species promote intestinal mucus production (Engevik et al. 2019; Gutierrez et al. 2023; Schroeder et al. 2018), suppress host inflammatory responses (Ojima et al. 2020; Engevik, Herrmann, et al. 2021; Sun et al. 2024; Okada et al. 2009; Xu et al. 2025) and exclude pathogens (Ronkainen et al. 2025; Collado et al. 2005; Ricci et al. 2022; Shao et al. 2024; Harnvoravongchai et al. 2025; Vazquez‐Gutierrez et al. 2016). *Bifidobacterium* species are also well known to participate in the gut‐brain axis, and administration of *Bifidobacterium* to mice and humans has been shown to alter brain chemistry and normalize behavior (Luck et al. 2021, 2020; Luk et al. 2018; Yang et al. 2017; Knox et al. 2025; Zhu et al. 2023; Wang et al. 2019; Tamayo et al. 2025; Kumar et al. 2024). Saffron has been shown to positively impact the gut‐brain axis (Pontifex et al. 2022; Lang et al. 2025; Cerdá‐Bernad et al. 2022; Lai et al. 2025) and it is possible that *Bifidobacterium* species may be contributing to these effects. Future in vivo studies in people would be valuable to better understand the association between saffron, *Bifidobacterium*, and improved brain function.

In addition to *Prevotella* and *Bifidobacterium*, we also found elevated levels of *Escherichia* in our bioreactors treated with saffron. The strain we used in this defined microbial community was *E. coli* Nissle 1917. *E. coli* Nissle 1917 is a widely studied commensal strain with probiotic properties and it has been shown to suppress intestinal inflammation and improve barrier function in mice and humans (Kruis et al. 2004; Altenhoefer et al. 2004; Zhao et al. 2022; Park et al. 2021; Chen et al. 2023; Schultz et al. 2004; Schultz 2008). Recent studies have demonstrated that saffron suppresses inflammation (Ashktorab et al. 2019, 2024; Banskota et al. 2021) and it is possible that commensal *E. coli* could participate in the anti‐inflammatory effects of saffron in the gut. It would be interesting in human studies to examine the synergy between *E. coli* Nissle 1917 and saffron administration in patients with IBD. It is possible that saffron may give *E. coli* Nissle a competitive advantage and may improve inflammatory outcomes.

Beyond effects on community structure, we found that saffron enhanced microbial communication through increased production of the quorum sensing molecule AI‐2. AI‐2 is broadly conserved across bacterial species and serves as a universal signal for interspecies communication (Zhang et al. 2020). Enhanced AI‐2 signaling facilitates cooperative metabolic interactions within a community. AI‐2 signaling has been well studied in many bacteria, where it controls gene expression, motility, and biofilm production (Rader et al. 2007; Li et al. 2017; Lee and Song 2005; Zhao et al. 2010; Ziegert et al. 2024). Interestingly, phenolic compounds have been shown to inhibit pathogens and reduce virulence (Santos et al. 2021; Lima et al. 2023; Higuera‐Ciapara et al. 2024; Helcman et al. 2025; Singh, Nair, et al. 2025). Little is known about the effects of polyphenols on quorum sensing in commensal bacteria. It is unclear how saffron impacts AI‐2 signaling, but it is possible that saffron‐derived polyphenols act as modulators of bacterial communication, either by enhancing signaling pathways that promote cross‐feeding and community stability or by selectively stimulating AI‐2 producers. More work will be needed to disentangle whether saffron directly amplifies AI‐2 synthesis, alters AI‐2 perception, or indirectly shapes signaling by changing community composition.

In this study, we found that saffron elevated the levels of the branched short chain fatty acid isobutyrate. Isobutryate can be produced via the fermentation of valine by *Blautia, Bacteroides* and *Prevotella* species (Tingler et al. 2025; Horvath et al. 2022). Administration of isobutyrate to pigs was shown to increase *Prevotella* abundance and elevate levels of indole‐3‐lactic acid (Fang et al. 2025). Consistent with these findings, we observed high levels of isobutyrate, *Prevotella*, and indole‐3‐lactic acid in our bioreactors treated with saffron, suggesting a potential positive feedback loop. In the same pig model, isobutyrate administration was associated with improved intestinal barrier‐related markers (Fang et al. 2025); suggesting that isobutyrate could positively impact the gut epithelium. We were surprised to find that saffron reduced the levels of propionate, butyrate, and valerate. A study by Singh et al. found that saffron supplemented in mice elevated 3‐aminoisobutyric acid, 2‐aminobutyric acid and 4‐hydroxybutyric acid (Singh et al. 2023). We did not measure these compounds in our targeted assay, but it is possible that these modified SCFAs could also be elevated in our cohort as well.

Another interesting finding in this study was that saffron redirected tryptophan catabolism toward neuroactive compounds. Specifically, we found that saffron reduced tryptophan levels while increasing the levels of tryptamine and indole‐3‐acetic acid. Tryptamine is structurally similar to serotonin and can activate serotonin receptors (Bhattarai et al. 2018, 2020). Activation of serotonin receptors has been linked to mucus secretion and improved barrier function (Bhattarai et al. 2020; Hoffman et al. 2012). Additionally, indole‐3‐acetic acid has been shown to activate aryl hydrocarbon receptors (AHRs) carbon receptors, limit inflammation and promote wound healing (Cao et al. 2025; Li et al. 2024; Kou et al. 2024; Ihekweazu et al. 2021). Saffron supplementation has been shown to reduce inflammation in animal models and in inflammatory bowel disease patients (Ashktorab et al. 2019, 2024; Rashid et al. 2024; Singh et al. 2023). It is possible that the beneficial effects of saffron could be due in part to tryptamine and indole‐3‐acetic acid production.

Similar to our findings with tryptophan, we found that saffron reduced tyrosine and L‐DOPA but markedly increased dopamine levels, demonstrating a clear push toward catecholamine production. Consistent with our findings, a previous mouse study also reported that saffron supplementation reduced intestinal tyrosine concentrations (Singh et al. 2023). Tyrosine can be converted to dopamine by tyrosine decarboxylases and these genes are only found in a few gut bacteria; one of which is *E. faecalis* . Tingler et al. (2025) recently demonstrated that *E. faecalis* in mono‐culture reduces tyrosine by 6 h of incubation and generates dopamine, with maximal production of dopamine occurring at 24 h. Since none of our other bacteria in the community harbor the tyrosine hydroxylase gene related to dopamine synthesis in their genome, we speculate that *E. faecalis* is the organism that is specifically responding to saffron with dopamine production. *E. faecalis* can also use tyrosine to generate tyramine (Tingler et al. 2025). However, in the bioreactor community, we did not observe any production of tyramine, suggesting that saffron selectively drives *E. faecalis* synthesis of dopamine.

We also found that saffron elevated GABA levels in our bioreactors. *Lactobacillus, Lactococcus, Bacteroides*, and *Bifidobacterium* species can all generate GABA from glutamate (Horvath et al. 2022; Luck et al. 2021; Pokusaeva et al. 2017; Cui et al. 2020; Yunes et al. 2016, 2020; Gomes et al. 2023; Laroute et al. 2023; Otaru et al. 2021; Tingler et al. 2025; Konstanti et al. 2023; Duranti et al. 2020; Wang et al. 2025; Strandwitz et al. 2019). These bacteria tend to produce GABA in order to reduce their intracellular pH and survive in acidic environments (Cui et al. 2020; Otaru et al. 2021; Sharafi et al. 2021). We did not measure the pH of the bioreactors, but it is possible that the saffron treated communities may have a lower pH that drives GABA production. GABA is an important neurotransmitter in the intestine, where it regulates mucus secretion, enteric nervous system signaling, and intestinal motility (Luck et al. 2021; Pokusaeva et al. 2017; Loeza‐Alcocer et al. 2019; Gros et al. 2021; Auteri et al. 2015; Deng et al. 2023; Liao et al. 2022; Ren et al. 2017). GABA producing bacteria have been correlated with reduced depression and anxiety (Strandwitz et al. 2019). Likewise, saffron has been associated with decreased depression (Chauhan et al. 2024; Jackson et al. 2020; Shafiee et al. 2018; Hausenblas et al. 2013). Since saffron increased the production of GABA in our defined communities, it is possible that GABA could be part of the pathway that reduces depression. However, more work, particularly work with GABA deficient microbes in germ‐free animals, will be necessary to dissect the role GABA plays in the positive attributes of saffron.

In this study, saffron also elevated the levels of formic acid. Formic acid is a one‐carbon fermentation product generated primarily from pyruvate via pyruvate formate lyase (PFL) under anaerobic conditions (Sawers 2025; Sawers and Clark 2004; Kammel et al. 2022). This pathway is characteristic of facultative anaerobes such as *Escherichia, Enterococcus*, and *Streptococcus* species, all of which were members of our defined community. The observed increase in formic acid, together with enhanced bacterial growth, suggests that saffron may promote fermentative metabolism and redox cycling within these taxa. Since formate production regenerates NAD^+^, sustaining glycolysis in oxygen‐limited environments, its accumulation may indicate a global shift toward reductive energy metabolism and heightened microbial metabolic activity. In the context of host physiology, elevated formic acid could have several implications. Formate can be absorbed into the circulation, where it contributes to systemic one‐carbon metabolism (Brosnan et al. 2018; Pietzke et al. 2020). These findings suggest that formic acid may serve as a potential biomarker of saffron‐induced microbial metabolic reprogramming.

Saffron is a complex botanical preparation that contains multiple bioactive compounds with distinct biological activities (Gutheil et al. 2012; Criado‐Navarro et al. 2024; Avila‐Sosa et al. 2022; Marrone et al. 2024). The major constituents include crocin and crocetin, carotenoid derivatives responsible for the characteristic color of saffron, as well as picrocrocin and safranal, which contribute to its bitter taste and aroma (Giaccio 2004; Bhat and Broker 1953; Abdullaev and Espinosa‐Aguirre 2004). Crocins are glycosylated carotenoids that can be hydrolyzed to crocetin in the gastrointestinal tract, and these compounds can influence oxidative stress, mitochondrial activity, and inflammatory signaling pathways (Zhao et al. 2021). Safranal and picrocrocin have also been associated with antioxidant effects (Frattaruolo et al. 2023; Esmaealzadeh et al. 2023; Rezaee and Hosseinzadeh 2013). Since saffron is composed of multiple bioactive compounds, it is difficult to determine which specific constituents are responsible for the microbial effects observed in this study. It is therefore likely that the phenotypes we observed reflect the combined and potentially synergistic actions of several saffron‐derived molecules rather than the activity of a single compound. Future studies using purified individual saffron components will be important to dissect the relative contributions of these molecules and to determine which constituents are primarily responsible for driving the microbial phenotypes described here.

Although our reductionist model provides a controlled framework for dissecting microbial‐dietary compound interactions, it has limitations. For example, the 14‐member consortium does not capture the full complexity of the human gut microbiota, and the absence of host cells, such as the epithelium, immune cells, and neurons, precludes direct assessment of host responses. Future studies in gnotobiotic animals will be needed to determine how saffron impacts complex microbiota in vivo, how microbial metabolites impact the host, and whether these changes contribute to saffron's reported health benefits. Additionally, human studies are necessary to fully understand the impact of saffron on the human gut microbiome and metabolome. In the future, we hope to explore the impact of saffron on microbial communities and their metabolites in healthy individuals and patients with intestinal disorders, such as IBD. It will be particularly interesting to identify the impact of saffron on dysbiotic communities and see if it still is able to maintain its pro‐health effects. Despite the limitations of our study, our findings establish that saffron exerts broad effects on microbial growth, communication, and metabolism, highlighting its potential as a dietary modulator of microbiota‐host interactions.

## Author Contributions

**Robert Proos:** investigation, methodology, resources, writing – review and editing. **Hassan Brim:** resources, investigation, writing – review and editing. **Ahmad Imran:** investigation, resources, writing – review and editing. **Santosh Kapil Kumar Gorti:** investigation, methodology, resources, writing – review and editing. **Makenna Grozis:** investigation, writing – review and editing. **Thomas D. Horvath:** investigation, formal analysis, resources, methodology, data curation, writing – review and editing, funding acquisition. **Adelaide E. Horvath:** conceptualization, investigation, writing – original draft, visualization. **Paul R. S. Baker:** investigation, methodology, resources, writing – review and editing. **Melinda A. Engevik:** data curation, resources, supervision, formal analysis, conceptualization, writing – review and editing, funding acquisition, visualization. **Hassan Ashktorab:** resources, supervision, formal analysis, conceptualization, writing – review and editing.

## Funding

This study was supported by South Carolina INBRE Research Experience for Undergraduates (AEH, MG), American Society for Investigative Pathobiology (ASIP) Summer Research Opportunity Program in Pathology (AEH, MG), S10OD036416 (TDH), P30DK056338 (Texas Medical Center Digestive Diseases Center; TDH), P20GM120457 (MAE), P30DK123704 (MAE), and R35GM155451 (MAE).

## Conflicts of Interest

T.D.H. is an Editorial Board Member and is contracted as an Associate Academic Editor for Cell Press—STAR Protocols. No other authors have anything to declare.

## Data Availability

All data generated or analyzed during this study are available from the corresponding author upon reasonable request.
